# Lactate Dehydrogenase as a Potential Therapeutic Drug Target to Control *Babesia bigemina*

**DOI:** 10.3389/fcimb.2022.870852

**Published:** 2022-04-19

**Authors:** Lan He, Reginaldo G. Bastos, Long Yu, Jacob M. Laughery, Carlos E. Suarez

**Affiliations:** ^1^ State Key Laboratory of Agricultural Microbiology, College of Veterinary Medicine, Huazhong Agricultural University, Wuhan, China; ^2^ Department of Veterinary Microbiology and Pathology, College of Veterinary Medicine, Washington State University, Pullman, WA, United States; ^3^ Animal Disease Research Unit, United States Department of Agriculture - Agricultural Research Service, Pullman, WA, United States

**Keywords:** babesiosis, *Babesia bigemina*, gossypol, lactate dehydrogenase (LDH), drug target, babesicidals

## Abstract

*Babesia bigemina* is a tick-borne apicomplexan hemoprotozoan responsible for bovine babesiosis. The current drugs used for bovine babesiosis treatment have several drawbacks, including toxicity, the lack of effectiveness to clear the parasite, and potential to develop resistance. Identifying compounds that target essential and unique parasite metabolic pathways is a rational approach toward finding alternative drug treatments. Based on the genome sequence and transcriptomics analysis, it can be inferred that anaerobic glycolysis is the dominant adenosine triphosphate (ATP) supply for *Babesia*, and lactate dehydrogenase (LDH) is one of the essential enzymes in this pathway. Furthermore, the *Babesia* LDH sequence is distinct from its bovine homologue and thus a potential chemotherapeutic target that would result in decreasing the ATP supply to the parasite but not to the host. Gossypol is a known efficient specific inhibitor of LDH in the *sensu stricto B. bovis* and the *sensu lato B. microti*, among other related parasites, but no such data are currently available in the *sensu stricto B. bigemina* parasites. Hereby, we show that the LDH amino acid sequence is highly conserved among *sensu stricto* but not in *sensu lato Babesia* spp. A predictive structural analysis of *B. bigemina* LDH showed the conservation of the key amino acids involved in the binding to gossypol compared to *B. bovis*. Gossypol has a significant (P < 0.0001) inhibitory effect on the *in vitro* growth of *B. bigemina*, with IC_50_ of 43.97 mM after 72 h of treatment. The maximum IC (IC_98_) was observed at 60 mM gossypol. However, a significant effect on the viability of cattle PBMC was observed when the cells were cultured with 60 mM (IC_98_) gossypol compared with DMSO-exposed control cells. Interestingly, *B. bigemina* cultured at 3% oxygen expresses significantly higher levels of LDH and is more resistant to gossypol than the parasites maintained at ambient conditions containing ~20% oxygen. Altogether, the results suggest the potential of gossypol as an effective drug against *B. bigemina* infection, but the risk of host toxicity at therapeutic doses should be further evaluated in *in vivo* studies.

## Introduction

Babesiosis is an emerging tick-borne hemoparasitic disease that poses an important threat to human and animal health. Bovine babesiosis is an economically important disease of cattle caused by the apicomplexan parasites *Babesia bovis* and *B. bigemina* in most tropical and semitropical regions worldwide (Ristic, 1988; Bock et al., 2004), and by other *Babesia* species, such as *B. divergens* in Europe (Zintl et al., 2003) and *B. orientalis* (He et al., 2021) and *B. ovata* in Asia (Yoshinari et al., 2013; He et al., 2021). While *B. bovis* has received much attention from the scientific community because it causes cerebral babesiosis and consequently high mortality, *B. bigemina* research has had less attention despite being highly pathogenic and widely spread and the fact that it can be transovarially transmitted by a wider range of *Rhipicephalus* ticks, including *Rhipicephalus microplus*, *R. annulatus*, and *R. decoloratus*, among others (He et al., 2021). The typical manifestations of acute *B. bigemina* infection include high fever, anorexia, severe anemia with hemoglobinuria and hemoglobinemia, and high parasitemia, among other signs (Hunfeld et al., 2008).

The control of bovine babesiosis is currently based on the use of live attenuated vaccines, babesicidal drugs, and tick control measures. The treatment of *B. bigemina*-infected animals usually involves the use of imidocarb dipropionate or diminazen aceturate (Vial and Gorenflot, 2006). However, these drugs cannot completely clear the parasite and may be toxic to the vertebrate hosts. In addition, there is the risk of the selection of drug-resistant parasites, causing relapse; therefore, alternative drugs are needed (Gray and Potgieter, 1981; Butler et al., 2008; Mosqueda et al., 2012). The approaches toward drug discovery usually include identifying unique parasite metabolic pathways or specific enzymes required for critical metabolic pathways. The glycolytic pathway has emerged as a possible target for drug development because of its essential role for the apicomplexan parasites as the main mechanism for the generation of adenosine triphosphate (ATP), an essential intermediate molecule that is required in most metabolic synthetic pathways in the life cycle of *B. bigemina*. Lactate dehydrogenase (LDH) is a critical enzyme of the glycolytic pathway and is present in most prokaryotic and eukaryotic cells (Rogatzki et al., 2015). Specifically, LDH catalyzes the conversion of pyruvate to lactate with the regeneration of NADH to NAD+ (**Figure S1**). This reaction is essential in hypoxic and anaerobic conditions when ATP production by oxidative phosphorylation is disrupted, causing an upregulation of the LDH activity. As mentioned above, the conversion of pyruvate to lactate results in the oxidation of the dinucleotide NADH to NAD+ recycling the glycolysis process, enabling ATP production, which is vital for cell survival and development. LDH is primarily expressed in the cytosol of the parasites, although it was also found to be associated with the internal side of host erythrocytes infected with *B. bovis* or *B. microti* (Bork et al., 2004; Vudriko et al., 2014; Yu et al., 2019). The overall structure of LDH is conserved in bovine and *Babesia*; however, there are important sequence and characteristic differences between these species that make this enzyme a possible target for pursuing alternative chemotherapeutic approaches for the control of bovine babesiosis (Yu et al., 2019).

Gossypol is an LDH activity inhibitor that has recently become the focus of research and a promising candidate for novel drug development against apicomplexan parasites, including *B. bovis* (Bork et al., 2004) and *B. microti* (Yu et al., 2019). Furthermore, Bork et al. (2004) demonstrated that gossypol could inhibit the enzymatic activity of recombinant *B. bovis* LDH and Vudriko et al. (2014) on the *B. microti* LDH. It is important to identify a pan-inhibitory drug that can control the main agents of bovine babesiosis, but it remains unknown whether gossypol can also inhibit the growth of *B. bigemina*. Ideally, such inhibition should occur at drug concentrations that are comparable to *B. bovis*, in order to be practical for the simultaneous treatment of coinfected animals, and below the threshold dose currently considered as toxic to bovines. Therefore, in this study, we explore the potential of gossypol to inhibit the development of *B. bigemina* in *in vitro* cultures. The results from this research set the rationale to further develop alternative drugs that can be effective to treat cattle infected with *B. bigemina* and thus contribute to expanding our arsenal of chemotherapeutics available for the control of bovine babesiosis.

## Materials and Methods

### *B. bigemina* Cultures

The Puerto Rico *B. bigemina* strain (Silva et al., 2020) was maintained in *in vitro* cultures (Vega et al., 1985) in 48-well plates containing 465 μl of culture per well, with 25 μl cattle red blood cells (RBCs) and 445 μl HL-1 medium (pH 7.2; 2.38 g/L N-2-Hydroxyethylpiperazine-N’-2-Ethanesulfonic Acid (HEPES), 5 ml/L L-glutamine, 60 U/ml of penicillin G, 60 μg/ml of streptomycin, and 0.15 μg/ml of amphotericin B; Sigma-Aldrich, St. Louis, MO, United States) supplemented with 20% bovine serum. The plate was incubated at 37°C in an atmosphere of 5% CO_2_. The culture was split every other day with 1:10 (46.5 μl was added into a new well).

### Multiple Alignments and Phylogenetic Analysis

The nucleotide sequence of *B. bigemina* lactase dehydrogenase (XM_012911368) (BbigLDH) was subjected to BLASTn using the GenBank^®^ database. Multiple related sequences from apicomplexan parasites and mammalian hosts, including *Homo sapiens* (AK298834), *Bos taurus* (NM_174099), and *Bubalus bubalis* (XM_006056061), were selected. Multiple alignments were performed by using MAFFT version 7. Neighbor-joining trees (Saitou and Nei, 1987) based on the selected nucleotide sequences were constructed using MEGA X (Kumar et al., 2018). The percentage of replicate trees in which the associated taxa clustered together in the bootstrap test (1,000 replicates) are shown next to the branches (Felsenstein, 1985). The tree is drawn to scale, with branch lengths in the same units as those of the evolutionary distances used to infer the phylogenetic tree. The evolutionary distances were computed using the maximum composite likelihood method (Tamura et al., 2004) and presented as units of the number of base substitutions per site. Codon positions included were 1st+2nd+3rd+noncoding. All ambiguous positions were removed for each sequence pair (pairwise deletion option).

### BbigLDH Structure Prediction

The amino acid sequence of BbigLDH shares a high degree of similarity to the reported *B. orientalis* LDH (BoLDH). For the structure-based study, the three-dimensional (3D) structure of BbigLDH was predicted by using a homology modeling method, and the 3D structure model was produced by using the SWISS-MODEL tool (https://swissmodel.expasy.org/interactive) according to the previously reported crystal structure of BoLDH (PDB code 7w8a). To reveal the binding mode of gossypol with BbigLDH, we performed a molecular docking and simulation using the predicting 3D model. Before running the CDOCKER program, the 3D homology model was further processed using the Discovery Studio 2018 Client, including deleting dopant atoms, original ligands, and water molecules; cleaning geometry; defining the active site and adding hydrogen atoms. The structure data format file of gossypol was downloaded from the chemical database (https://pubchem.ncbi.nlm.nih.gov) under the PubChem CID 24895349.

### Flow Cytometric Analysis of *B. bigemina* Growth in *In Vitro*

The parasitemia of *B. bigemina* cultures was determined by flow cytometry using a Guava^®^ easyCyte flow cytometer (Luminex Corp., Austin, TX, United States). Briefly, 3 μl mixed culture was added into 200 μl phosphate buffer solution (PBS), centrifuged at 350 × g for 3 min at 4°C. The supernatant was discarded, and the pellet was resuspended with 200 μl PBS containing 5 μM hydroethidine (HE) (Invitrogen, Carlsbad, CA, USA). Cells were incubated with HE at 37°C for 20 min in the dark. The plate was centrifuged as described above, the supernatant was discarded, and the pellet was washed with 200 μl PBS one time. Then, the pellet was suspended in 200 μl PBS; 35 μl of each well was added into a new well containing 200 μl PBS and then analyzed by flow cytometry at a ratio of 500–800 cells/μl with 10,000 events collected. The results were analyzed by FlowJo™ v10 Software (BD Life Sciences, Ashland, OR, USA). Uninfected normal cattle RBCs were used as negative control for the flow cytometric analysis.

### Gossypol IC_50_ Calculation

*B. bigemina* was cultured until the parasitemia reached 3%. The culture was harvested and diluted to final parasitemia 0.2% with culture medium, and then cultured in 96-well plates (200 ml/well total volume). Gossypol was then added immediately at different concentrations (0, 1, 10, 20, 30, 40, 50, 60, and 100 mM), and the approximate IC_50_ was calculated. In order to obtain more accurate IC_50_, additional concentrations (0, 40, 41, 42, 43, 44, 45, 46, 47, 48, 49, 50, and 100 mM) were tested against *B. bigemina* in *in vitro* culture. Uninfected normal cattle RBCs were used as blank controls. All treatments were set up in triplicates. The parasite culture medium was changed every 24 h, and parasitemias were checked at 72 h after treatment by flow cytometric analysis.

### Cytotoxicity Assays of Cattle Peripheral Blood Mononuclear Cell

The cytotoxicity of gossypol in cattle peripheral blood mononuclear cells (PBMCs) was examined using 60 mM of the drug, which corresponds to the maximum inhibitory concentration (IC_98_) in *B. bigemina*, as shown in the current study. Cattle PBMCs were isolated by standard protocols. Briefly, blood was collected from *Babesia-*free cattle *via* jugular venipuncture into BD Vacutainer™ ACD (acid citrate dextrose) with solution A (BD, United States). PBMCs were isolated by Histopaque^®^ (Sigma Life Science, United States) gradient and suspended in complete RPMI-1640 (cRPMI-1640) (25 mM HEPES, without L-glutamine [Sigma Life Science, United States], 10% bovine serum [collected from donor animal], 2 mM L-glutamine [Sigma Life Science, United States], 1 μg/ml gentamycin [Fluka Biochemika, United States], 50 μM 2-mercaptoethanol [Merck, United States]). One hundred microliters of PBMCs (2 × 10^4^ cells) were added into 96-well plates and treated with 60 mM gossypol or 2.5 μg/ml concanavalin A (ConA) (Invitrogen, United States). Considering that gossypol was diluted in dimethyl sulfoxide (DMSO), 0.06% DMSO, which corresponds to the amount present in 60 mM gossypol, was used as negative control. PMBCs without any treatment were used as a blank control. All treatments were performed in triplicates. The viability of cattle PBMCs was evaluated by monitoring cell metabolic activity using the Water Soluble Tetrazolium Salt (WTS-1) colorimetric assay (Roche Applied Science, Branford, CT, USA), following the manufacturer’s protocol. PBMC viability was examined at 24, 48, and 72 h after gossypol exposure. Absorbance at 440 nm was measured using an enzyme linked immunosorbent assay (ELISA) plate reader at 4 h after adding the WST-1 reagent.

### Gossypol Effect on *B. bigemina* Growth

*In vitro* growth inhibition assays were carried out over a 9-day period using initial PPE (percent of parasitized erythrocytes) of 0.2% and 2% under high (5% CO_2_ incubator) or low oxygen concentrations (3% O_2_, 5% CO_2_, and 92% N_2_). Parasites were grown in the presence of 43.97 mM (IC_50_) and 60 mM (IC_98_) gossypol. Diminazene aceturate (DA) at a concentration of 5 mM (Selleckchem, Houston, TX, USA) was used as a drug control to inhibit the parasite growth. Parasite cultures in a medium without any treatment and non-infected RBCs were used as blank controls. The culture medium (150 ml) was changed at days 1 and 2 (72 h treatment in total) with representative treatments, respectively. Starting at day 3, 150 ml fresh medium without gossypol or DMSO was changed every 24 h. Plates were split at days 3, 5, and 7, and parasitemia was monitored by flow cytometer at days 1, 2, 3, 8, and 9.

### LDH Transcription Levels in *In Vitro* Cultured *B. bigemina*

*B. bigemina* was cultured under ambient oxygen (approx. 20% O_2_ and 5% CO_2_) or low oxygen (3% O_2_, 5% CO_2_, and 92% N_2_) conditions. Samples were collected until parasitemia reached 5%. RNA was extracted by using Trizol (*Invitro*gen, Waltham, MA, USA). RNA purity and concentration were monitored by using a Nanadrop 2000 (Thermofisher, Waltham, MA, USA). Five hundred nanograms of RNA was used for cDNA synthesis by using the SuperScript™ First-Strand kit (*Invitrogen*, Waltham, MA, USA).

Three pairs of primers were designed based on the sequence of BbigLDH and 18S rRNA by using RT-PCR design wizard in Clone manager version 8, respectively. All the primers were subjected to quantitative polymerase chain reaction (qPCR); the pairs showed stable results, and the melt curve was selected for further study. The LDH transcription level was determined by qPCR, and the *B. bigemina* 18S rRNA gene was used as a reference. The primers for qPCR are listed in **Table 1**.

**Table 1:** qPCR primers used in this study.

Primer	Sequence
**qBbgLDH-F**	ACGTCAAGGTCAACGGACTC
**qBbgLDH-R**	CGATGGTGCGCTTCTCAATG
**q18S-F**	AGCGTGGGGGAAGTTTAAGG
**q18S-R**	TTCGGGACAGGACAAACTCG

### Statistical Analysis

Statistical analysis was performed using GraphPad Prism v.7 (GraphPad Software, Inc.). Gossypol IC_50_ was calculated by using the dose-inhibition model. The *B. bigemina* growth assay and cytotoxicity assay data were analyzed with two-way ANOVA.

## Results

### LDH Is a Potential Drug Target for *B. bigemina*

**Figure 1:**
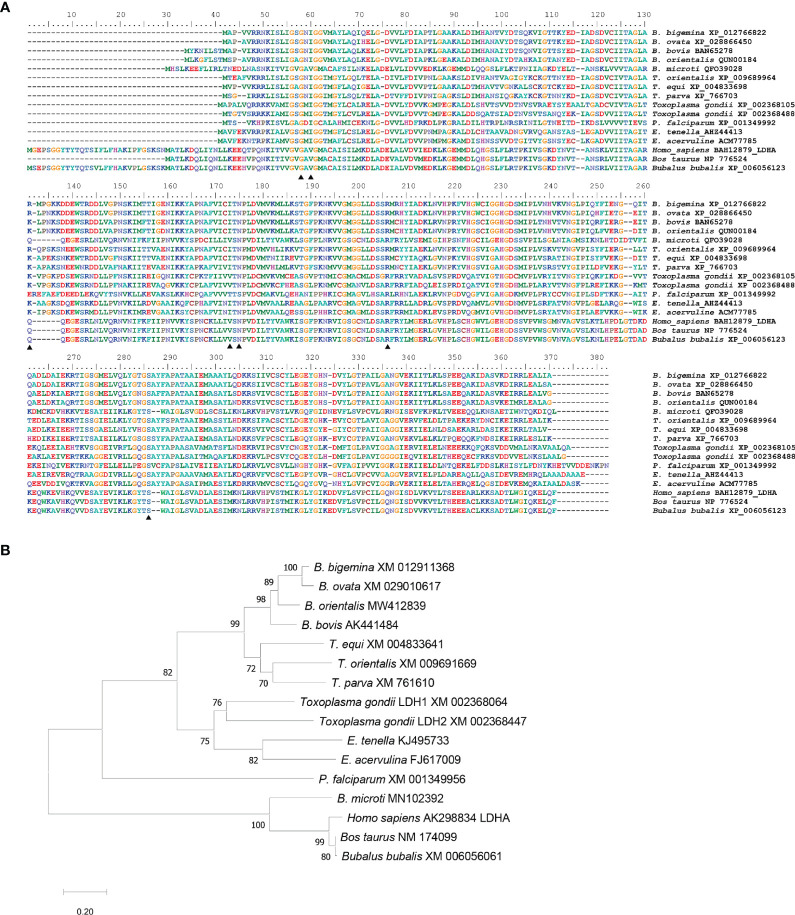
Multiple alignments and phylogenetic analysis. **(A)** Multiple alignments of LDH amino acid sequence by MAFFT version 7. Thirteen apicomplexan parasites sequences and three mammalian host sequences were compared. GenBank accession numbers were followed after species names. B, *Babesia*; T, *Theileria*; P, *Plasmodium*; E, *Eimeria*. Similar amino acids are shown in gray; identical amino acids in black triangles indicated the predicted residuces for inhibitor gossypol. **(B)** Phylogenetic analysis of LDH nucleotide sequences from 13 apicomplexan parasites and 3 mammalian hosts. The optimal tree is calculated by neighbor-joining with bootstrap estimates from 1,000 replicates. Evolutionary distances showed with the scale with branch lengths in the same units infer the phylogenetic tree.

The *B. bigemina* LDH gene encodes for a protein of 382 amino acids. Multiple comparative alignments of amino acid sequences of LDH among apicomplexan and vertebrate species demonstrate that this protein is highly conserved between apicomplexan parasites but significantly different compared with mammalian hosts, including *B. taurus*, *B. bubalis*, and *H. sapiens* (**Figure 1A**). The LDH similarities ranges from 95.3% to 64.2% among Piroplasmida members. Interestingly, *B. microti* has a host-like LDH gene that has 65.3% identity with human LDH but only 25.8%, 27%, and 26.4% identity to the LDH of *B. bigemina*, *B. bovis*, and *B. orientalis*, respectively (**Table S1**) (Yu et al., 2019). The similarity between *B. bigemina* and mammalian host LDH amino acid sequences, including *B. bubalis*, ranges from 26.5% to 29.1% (**Table S1**). To further analyze these similarities, we constructed a phylogenetic tree using MEGA X (Kumar et al., 2018) with the sequences of multiple LDH genes derived from distinct Apicomplexa and mammalian species (**Figure 1B**). The optimal unrooted neighbor-joining tree showed that BbigLDH falls into the *Babesia* clade and closer to *B. ovata* (XM_029010617), while the mammalian host LDHs fall into a different clade with a highly significant bootstrap value (**Figure 1B**). Consistent with previous observations, the *B. microti* LDH gene clusters in the same clade as the mammalian LDH genes. Taken together, these sequence analysis comparisons set the rationale for testing whether gossypol, a well-known potent inhibitor of LDH in *B. bovis* and *B. microti*, can be considered as an alternative drug for treating cattle infected with *B. bigemina*.

### BbigLDH Active Pocket Is Highly Similar to BoLDH

**Figure 2:**
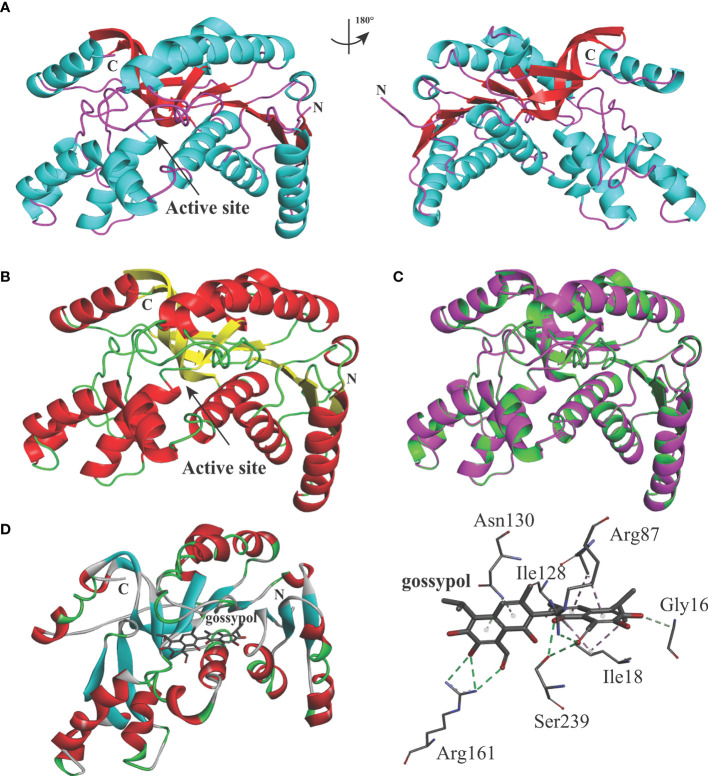
Structure models of *B. bigemina* LDH. **(A)** The 3D structure model of *B. bigemina* LDH. The 3D model was constructed by using SWISS-MODEL based on the reported apo BoLDH (7w8a). **(B)** BoLDH crystal structure. The structure of apo BoLDH was displayed as the cartoon, and the active pocket was labeled. **(C)** Structural comparison between apo BoLDH and BbigLDH (1t2d). BbigLDH (green) was superimposed on BoLDH without ligands (magenta). **(D)** Structural cartoon of BbigLDH with gossypol and oxalate on LDH catalytic pocket. The distribution of hydrogen bonds and hydrophobic interactions at the gossypol interface are shown. The interacting amino acids in the NADH binding pocket are labeled and shown as stick diagrams, and the dashed lines represent the hydrogen bonds and hydrophobic interactions between the respective donor and acceptor atoms. Gossypol was displayed as a stick diagram, respectively.

The predicted BbigLDH 3D model includes a cofactor binding site for NADH or NAD^+^ and a substrate binding site for pyruvate or lactate, and the secondary structure of BbigLDH was composed of nine α-helices, nine β-strands, and several loops (**Figure 2A**). Compared to the apo BoLDH structure (PDB accession no. 7w8a), we observed that the structures of BbigLDH and BoLDH are fully conserved as their amino acid sequence has a high identity of 84.42% (**Figures 2B, C**). For simulating the binding of BbigLDH and gossypol, the compound gossypol was docked into the 3D structure of BbigLDH using the software of Discovery Studio 2018 Client. In the binding model, we observed that seven amino acid residues, Gly16, Ile18, Arg87, Ile128, Asn130, Arg161, and Ser239, which are present in the catalytic pocket of BbigLDH, were predicted to be involved in the interaction with gossypol (**Figure 2D**). The seven residues were indicated in **Figure 1A** with black triangles.

### Gossypol Inhibits the Growth of *B. bigemina* in *In Vitro* Cultures

**Figure 3:**
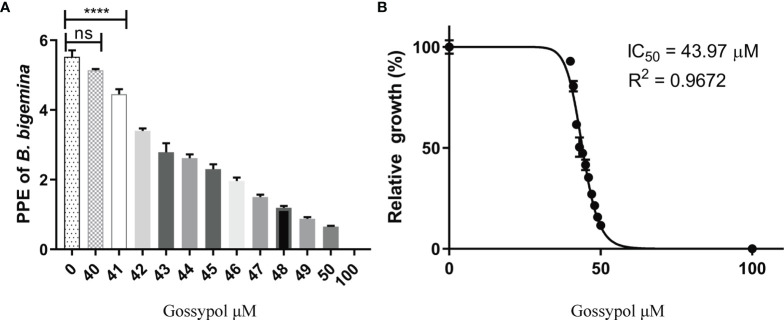
Relative growth of different concentration gossypol treated *B. bigemina*. **(A)** Gossypol efficacy in the growth of *B. bigemina in vitro* culture. Parasites were cultured at 5% cattle RBC until parasitemia is more than 3%. The cultures were diluted to 0.2% PPE and cultured with a total volume of 200 ml/well in 96-well plate in 5% CO_2_, treated with gossypol at different concentrations for 72 h. **(B)** IC_50_ of gossypol in *B. bigemina in vitro* culture analyzed by Graphpad Prism7 with dose-inhibition calculation. ns represents no difference; Four asterisks (****) represent statistically significant differences and P < 0.0001. ns, no significant difference.

The effect of gossypol on the growth of *B. bigemina in vitro* was tested by adding different concentrations of the drug into the parasite cultures with 0.2% initial PPE. The calculated parasitemias after 72 h posttreatment were 5.53%, 5.14%, 4.45%, 3.40%, 2.79%, 2.62%, 2.30%, 1.96%, 1.50%, 1.19%, 0.88%, 0.65%, and 0.01% at gossypol concentrations of 40, 41, 42, 43, 44, 45, 46, 47, 48, 49, 50, and 100 mM, respectively (**Figure 3**). The parasitemia was significantly lower (P < 0.0001) at 41 mM gossypol (4.45% PPE) than in negative control (5.53% PPE), while there is no significant difference (P = 0.1866) between 40 mM and negative control (**Figure 3B**). In addition, the data indicate that gossypol efficacy is dose dependent and that the drug inhibited 98% *B. bigemina* growth at 60 mM (IC_98_) and 50% at 43.97 mM (IC_50_) when compared to the negative control (**Figure 3B**).

### Effect of Gossypol on Cattle PBMC

**Figure 4:**
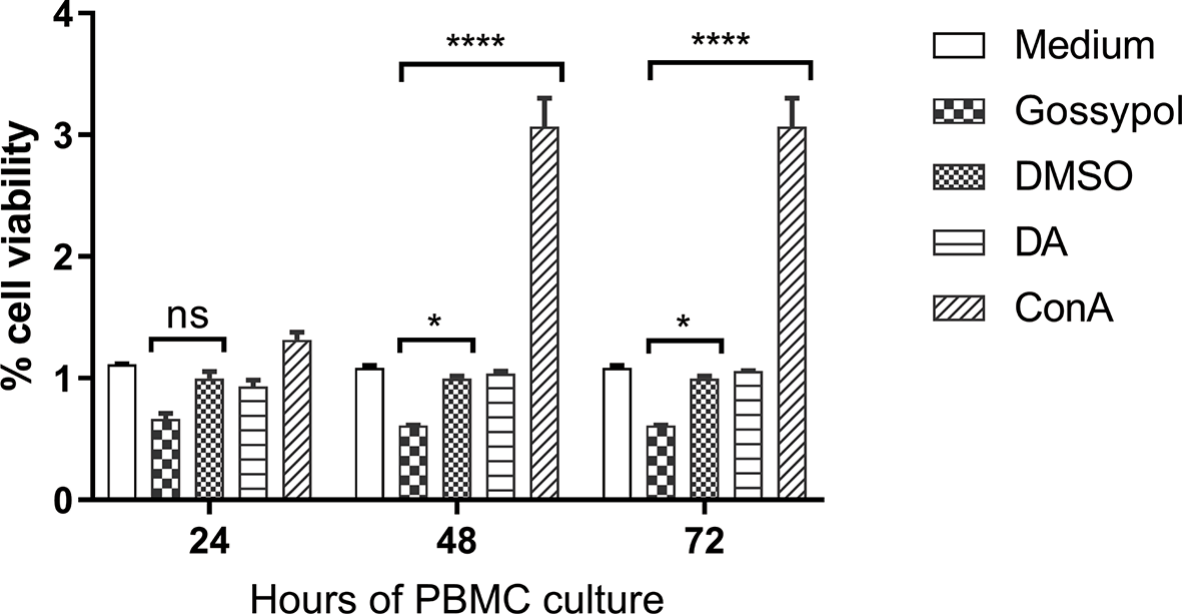
Cell viability over a period of 72 h after incubation with the IC_98_ of gossypol. PBMCs were obtained from cattle. Medium: PBMC cultured without adding compound, used as blank control; gossypol: PBMC cultured with 60 uM gossypol; DMSO: PBMC cultured with 0.06% DMSO was used as a negative control; DA: PBMC cultured with 5 μM diminazene aceturate as drug control; ConA: concanavalin A (ConA) was used as a positive control for cell proliferation. ns represents no difference; One asterisk (*) represent statistically differences and P < 0.0332; Four asterisks (****) represent statistically significant differences and P < 0.0001.

A cytotoxic assay using cattle PBMCs was performed to test the effect of gossypol on the viability of mammalian host cells. The cytotoxic assay was conducted at IC_98_ of gossypol (60 mM), using 0.06% DMSO as a negative control. The results showed that PBMC viability was not affected by the presence of gossypol compared with DMSO at 24 h (P = 0.0961), but significant inhibition was found at 48 h (P = 0.0376) and 72 h (P = 0.0376). In addition, significant cell proliferation was observed in PBMCs exposed to ConA for 2 days (P < 0.0001) and 3 days (P < 0.0001) in comparison to controls (**Figure 4**), indicating sufficient sensitivity of the WST-1 assay. Collectively, the results demonstrate that gossypol negatively affected the viability of bovine PBMCs *in vitro*, an aspect that should be taken into consideration for future *in vivo* studies.

### Gossypol Efficacy on Cultures of *B. bigemina* Depends on the Oxygen Concentration

**Figure 5:**
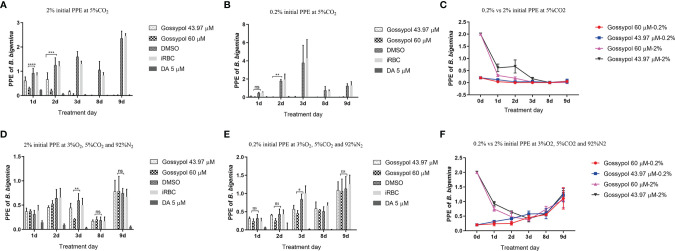
*B. bigemina* cultured with IC_50_ or IC_98_ gossypol in low oxygen (3% O_2_, 92% N_2_, and 5% CO_2_) or ambient oxygen (5% CO_2_) incubators. **(A)** *B. bigemina* cultured at ambient oxygen with 2% initial PPE; **(B)** *B. bigemina* cultured at ambient oxygen with 0.2% initial PPE; **(C)** Comparing *B. bigemina* cultured at ambient oxygen with 2% and 0.2% initial PPE. **(D)** *B. bigemina* cultured at low oxygen with 2% initial PPE; **(E)** *B. bigemina* cultured at low oxygen with 0.2% initial PPE; **(F)**: Comparing *B. bigemina* cultured at low oxygen with 2% and 0.2% initial PPE. Data were analyzed by using Graphpad Prism7 with two-way ANOVA. ns represents no difference; *p < 0.0332, **p < 0.0021, ***p < 0.0002, ****p < 0.0001.

Because LDH is an essential enzyme in the anaerobic glycolysis pathway, we hypothesized that the inhibitory gossypol efficacy in *B. bigemina* is associated to the oxygen concentration. To test this hypothesis, *B. bigemina* was cultured at ambient oxygen (5% CO_2_ incubator) and low oxygen (3% O_2_, 5% CO_2_, and 92% N_2_) with 0.2% or 2% initial PPE, respectively, in the presence or absence of gossypol. Results showed that the parasitemia of gossypol-treated cultures was significantly lower (P < 0.0001) compared to that in the DMSO control starting at day 1 with 2% PPE initial at ambient oxygen (**Figure 5A**). Compared to the 0.2% PPE at high oxygen, the parasitemia of 43.97 and 60 mM gossypol were only 0.123% and 0.04% at day 1, respectively, and the parasitemia of 0.06% DMSO control was 0.477% at day 1. However, statistical analysis showed no significant difference between treatment and control (gossypol 43.97 mM vs. DMSO, P = 0.9476; gossypol 60 mM vs. DMSO, P = 0.8931) (**Figure 5B**). Gossypol 43.97 mM vs. DMSO had a significant difference at day 2 (P = 0.0056) and day 3 (P < 0.0001) with 0.2% initial PPE at ambient oxygen (**Figure 5B**). After splitting the culture three times, both 43.97 and 60 mM gossypol fully eliminated the parasites regardless of the initial parasitemia (**Figure 5C**).

**Figure 6:**
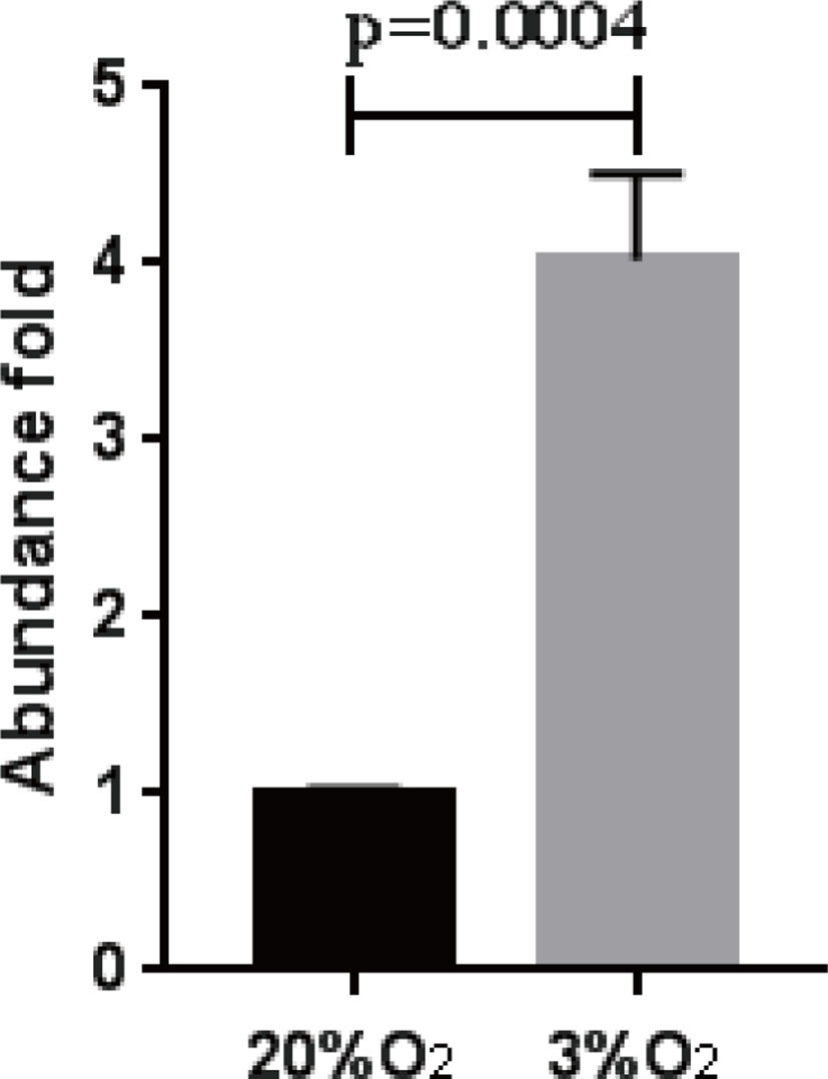
*B. bigemina* LDH abundance in different oxygen concentrations. *B. bigemina* was cultured in 20% and 3% oxygen, respectively.

When *B. bigemina* was cultured at low oxygen conditions (3% O_2_, 5% CO_2_, and 92% N_2_), the parasites survived regardless of 0.2% or 2% initial PPE with 60 mM gossypol, showing significant difference (P = 0.0017 for 2% initial PPE, and P = 0.0175 for 0.2% initial PPE) at day 3 (**Figures 5D, E**). However, there was no difference after splitting the culture three times (**Figure 5F**) with P-values 0.9933 and 0.9816 for 2% and 0.2% initial PPE, respectively. Taken together, the results indicate that *B. bigemina* has a much higher resistance to gossypol when developing in low oxygen conditions compared to ambient oxygen conditions. This may be due to the fact that the LDH expression level is much higher in low oxygen conditions than high oxygen conditions. To test this possibility, we examined the transcription level of *B. bigemina* LDH by qPCR. A significant (P=0.0004) upregulation of LDH transcripts was observed in *B. bigemina* cultures at 3% oxygen, compared to parasite cultures at ambient oxygen conditions (**Figure 6**).

## Discussion

Babesiosis is a threat to public health and food security worldwide. Bovine babesiosis, an economically important disease of bovine, negatively affects the cattle industry, especially in tropical and subtropical regions. The recent expansion of tick populations that transmit *Babesia* parasites has even further impacted the effect of the disease on cattle production (He et al., 2021). The treatment and control of animal babesisosis is currently based on the use of imidocarb dipropionate and diminazene aceturate (Vial and Gorenflot, 2006). However, these drugs are associated with several side effects and usually cannot eliminate the parasites from the hosts (Gray and Potgieter, 1981; Krause et al., 2000; Butler et al., 2008), and drug resistance has been described (Mosqueda et al., 2012). In fact, the possible development of *B. bigemina* resistance to currently available drugs would create a shortage of available treatments. Consequently, alternative drugs are needed.

In the present study, BbigLDH was evaluated as a potential drug target in *in vitro* cultures. A comparative sequence analysis of amino acid sequences revealed high identity between the BbigLDH and homologues from other protozoan parasites, such as *B. bovis* (Bork et al., 2004), *Plasmodium berghei* (Winter et al., 2003), and *Toxoplasma gondii* (Winter et al., 2003) but significant differences with vertebrate host LDH. The predicted structure of BbigLDH showed that the active pocket is highly similar to that of BoLDH. This observation suggests that the biological functions or the catalytic mechanisms of protozoan LDHs might not be identical as the mammalian hosts’ LDHs, and these differences could be used as targets for drug development purposes.

LDH is one of the most highly expressed enzymes by *Plasmodium falciparum* (Vander Jagt et al., 1990) and *B. microti* (Yu et al., 2019) of the glucose metabolic pathway. This enzyme is essential for completing the anaerobic life cycle, and compounds such as gossypol and its derivatives, which inhibit the enzyme activity, could potentially kill the parasite (Razakantoanina et al., 2000; Guo et al., 2020; Yu et al., 2020). Gossypol is a natural phenol derived from seeds of the cotton plant (Wu, 1989). The biological activities of gossypol, including contraceptive (Wu, 1989), anticancer (Keshmiri-Neghab and Goliaei, 2014; Rao and Narechania, 2016), and antimalarial (Razakantoanina et al., 2000), are known to occur by inhibiting the binding of reduced NADH to LDH (Conners et al., 2005; Yu et al., 2019) (**Figure S1**). The anti-parasitic efficacy of gossypol has also been investigated against *B. microti* and *B. bovis*, and LDH was confirmed as a potential therapeutic target for the drug (Razakantoanina et al., 2000; Bork et al., 2004; Yu et al., 2019). However, there is no previous report regarding the effect of gossypol against *B. bigemina*.

In order to test the efficacy of gossypol in *B. bigemina*, the IC_50_ was determined by using an *in vitro* culture of the parasite. The concentrations of gossypol required to fully inhibit the *in vitro* growth of *B. bigemina* in 20% oxygen (IC_50_ 43.97 mM, IC_98_ 60 mM) are similar to the concentrations required for *B. bovis* (Bork et al., 2004) and *Trypanosoma cruzi* (Montamat et al., 1982). This is of importance because the coinfections of *B. bovis* and *B. bigemina* are frequent in endemic areas of bovine babesiosis, and drug treatments would likely require similar dosages to efficiently control both parasites. However, the cell viability assays performed using cattle PBMCs demonstrated that gossypol also affects the viability of PBMCs in *in vitro* assays at the concentration of 60 mM (IC_98_). A previous study showed that gossypol had no effects on superovulation response or embryo development in pregnant cows (Velasquez-Pereira et al., 2002). In non-ruminant animals, a high concentration of gossypol may cause death by reducing the oxygen-carrying capacity of blood and hemolysis of RBCs (Vander Jagt et al., 2000; Gadelha et al., 2014). It will thus be necessary to perform *in vivo* experiments to evaluate the efficacy of gossypol *via* parenteral against *B. bigemina* and to determine whether gossypol is toxic for cattle and buffalo when administrated at *B. bigemina* inhibitory doses. Interestingly, when *B. bigemina* was cultured under low oxygen conditions, 60 mM significantly inhibited the growth of the parasites. Remarkably, this inhibition was reversible, and parasites eventually reemerged after splitting the cultures three times. However, qPCR analysis showed a 4-fold upregulation of the LDH expression level of *B. bigemina* cultured in low oxygen compared to ambient oxygen conditions, which suggests that adjustments in the doses of gossypol may be needed to efficiently inhibit LDH activity according to the oxygen condition.

Taken together, the data in this study suggest that the LDH of *B. bigemina* is a druggable target and gossypol could be considered a potential candidate as a babesicidal drug. Importantly, the IC_50_ of gossypol for *B. bigemina* was similar to the one for *B. bovis*, which are usually found as coinfecting agents in endemic areas. Remarkably, the inhibitory effect of gossypol on *B. bigemina* was dependent on the concentration of oxygen, and LDH transcription levels in the parasite can also be regulated by the level of oxygen in the environment. Future work will require an *in vivo* assessment of gossypol’s potential as an effective drug against *B. bigemina* infection and the risk of host toxicity at therapeutic doses.

## Data Availability Statement

The original contributions presented in the study are included in the article/**Supplementary Material**. Further inquiries can be directed to the corresponding author.

## Ethics Statement

The animal study was reviewed and approved by Institutional Animal Care and Use Committee of the University of Idaho, USA (IACUC Protocol #2013-66).

## Author Contributions

LH, RB, and CS designed the study and wrote the draft of the manuscript. LY performed the prediction structure of BbigLDH. LH and JL performed the experiments and analyzed the results. All authors have read and approved the final manuscript.

## Funding

The work was funded by the United States Department of Agriculture-Agriculture Research Service, CRIS Project No. 2090-32000-039-00D, and the USDA National Institute of Food and Agriculture (NIFA) (Award Number: 2020-67015-31809, Proposal Number: 2019-05375, Accession Number: 1022541), the National Natural Science Foundation of China (32002308), the Fundamental Research Funds for the Central Universities, China (Grant No. 2662019PY001).

## Conflict of Interest

The authors declare that the research was conducted in the absence of any commercial or financial relationships that could be construed as a potential conflict of interest.

## Publisher’s Note

All claims expressed in this article are solely those of the authors and do not necessarily represent those of their affiliated organizations, or those of the publisher, the editors and the reviewers. Any product that may be evaluated in this article, or claim that may be made by its manufacturer, is not guaranteed or endorsed by the publisher.

## Supplementary Material

Supplementary Figure 1 The schematic of lactate dehydrogenase catalyzed the reversable reaction of pyruvate to lactate.

## Abbreviations

LDH, lactate dehydrogenase; PPE, percent parasitized erythrocytes; BbigLDH, *Babesia bigemina* LDH; BoLDH, *Babesia orientalis* LDH.
