# Supplementary figures and images for: Lactate Dehydrogenase as a Potential Therapeutic Drug Target to Control Babesia bigemina
Source: Front Cell Infect Microbiol. 2022 Apr 19;12:870852. doi: 10.3389/fcimb.2022.870852 (PMC9062099; doi:10.3389/fcimb.2022.870852)

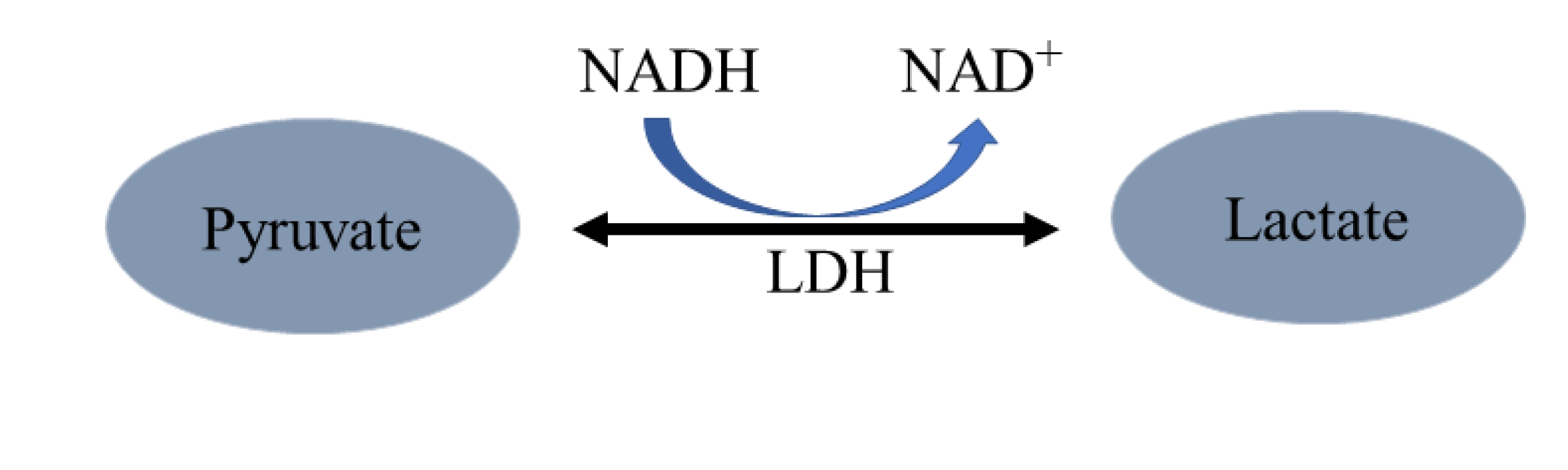

Supplement: Supplementary Figure 1 — The schematic of lactate dehydrogenase catalyzed the reversable reaction of pyruvate to lactate. [file Image_1.tif]
