# Supplementary material for: Lactate Dehydrogenase as a Potential Therapeutic Drug Target to Control Babesia bigemina
Source: Front Cell Infect Microbiol. 2022 Apr 19;12:870852. doi: 10.3389/fcimb.2022.870852 (PMC9062099; doi:10.3389/fcimb.2022.870852)
Supplement: Supplementary file 2 [file Table_1.docx]

| Seq-> | *B bigemina* | *B ovata* | *B bovis* | *B orientalis* | *T orientalis* | *T parva* | *T equi* | *Toxoplasma gondii* | *Toxoplasma gondii* | *E tenella* | *E acervulina* | *P falciparum* | *B microti* | *H*  *sapiens* | *Bos taurus* | *Bubalus bubalis* |
| --- | --- | --- | --- | --- | --- | --- | --- | --- | --- | --- | --- | --- | --- | --- | --- | --- |
| *B bigemina* | ID | 0.953 | 0.836 | 0.821 | 0.696 | 0.642 | 0.692 | 0.465 | 0.481 | 0.456 | 0.484 | 0.367 | 0.258 | 0.254 | 0.291 | 0.265 |
| *B ovata* |  | ID | 0.848 | 0.839 | 0.69 | 0.639 | 0.677 | 0.468 | 0.484 | 0.468 | 0.487 | 0.37 | 0.258 | 0.249 | 0.285 | 0.26 |
| *B bovis* |  |  | ID | 0.9 | 0.654 | 0.642 | 0.681 | 0.468 | 0.486 | 0.459 | 0.497 | 0.379 | 0.27 | 0.254 | 0.285 | 0.26 |
| *B orientalis* |  |  |  | ID | 0.651 | 0.648 | 0.687 | 0.465 | 0.48 | 0.451 | 0.488 | 0.381 | 0.264 | 0.262 | 0.294 | 0.268 |
| *T orientalis* |  |  |  |  | ID | 0.761 | 0.727 | 0.437 | 0.463 | 0.441 | 0.451 | 0.363 | 0.264 | 0.246 | 0.276 | 0.257 |
| *T parva* |  |  |  |  |  | ID | 0.704 | 0.449 | 0.487 | 0.444 | 0.46 | 0.38 | 0.267 | 0.241 | 0.264 | 0.243 |
| *T equi* |  |  |  |  |  |  | ID | 0.452 | 0.475 | 0.465 | 0.472 | 0.385 | 0.244 | 0.241 | 0.258 | 0.235 |
| *Toxoplasma gondii LDH1* |  |  |  |  |  |  |  | ID | 0.717 | 0.542 | 0.592 | 0.366 | 0.27 | 0.252 | 0.276 | 0.252 |
| *Toxoplasma gondii LDH2* |  |  |  |  |  |  |  |  | ID | 0.564 | 0.609 | 0.389 | 0.263 | 0.258 | 0.289 | 0.264 |
| *E tenella* |  |  |  |  |  |  |  |  |  | ID | 0.706 | 0.388 | 0.239 | 0.253 | 0.28 | 0.256 |
| *E acervulina* |  |  |  |  |  |  |  |  |  |  | ID | 0.403 | 0.266 | 0.256 | 0.281 | 0.256 |
| *P falciparum* |  |  |  |  |  |  |  |  |  |  |  | ID | 0.224 | 0.215 | 0.235 | 0.217 |
| *B microti* |  |  |  |  |  |  |  |  |  |  |  |  | ID | 0.653 | 0.695 | 0.642 |
| *Homo sapiens* |  |  |  |  |  |  |  |  |  |  |  |  |  | ID | 0.861 | 0.922 |
| *Bos taurus* |  |  |  |  |  |  |  |  |  |  |  |  |  |  | ID | 0.911 |
| *Bubalus bubalis* |  |  |  |  |  |  |  |  |  |  |  |  |  |  |  | ID |

Table S1 LDH amino acid sequence identity in selected *Babesia* species and host.
